# A narrative review of Neonatal Echocardiography Simulator Training (NEST): role of simulators in acquiring neonatal echocardiography skills effectively

**DOI:** 10.1038/s41390-025-04321-5

**Published:** 2025-08-12

**Authors:** Yogen Singh, Rangasamy Ramanathan, Mahmood Ebrahimi, Shahab Noori

**Affiliations:** 1 Department of Pediatrics, Division of Neonatology, University of California, UC Davis Children’s Hospital, Sacramento, CA USA; 2 Department of Pediatrics, Division of Neonatology, Cedars Sinai Guerin Children’s, Cedars Sinai Medical Center, Los Angeles, CA USA; 3 Fetal and Neonatal Institute, Division of Neonatology, Children’s Hospital Los Angeles, Department of Pediatrics, Keck School of Medicine, University of Southern California, Los Angeles, CA USA

## Abstract

**Abstract:**

Neonatologist performed echocardiography (NPE) is fast becoming as an essential tool to provide high-quality patient care in the neonatal intensive care units (NICU), especially to infants with hemodynamic instability. With limited training opportunities in the neonatal intensive care setting, learning echocardiography and implementation of published neonatal echocardiography training guidelines remain a challenge for the vast majority of neonatologists across the world. Hence, these guidelines have recommended the use of simulators for the initial echocardiography training. In the last two decades, simulators dedicated to developing skills in neonatal echocardiography have been developed and their effectiveness in imaging acquisition and interpretation has been validated. This manuscript explores the role of simulators in developing echocardiography skills, their pros and cons, available tools for the neonatologists and how they can utilize these tools in their training.

**Impact:**

- Neonatal echocardiography simulators have been validated as an effective tool in improving echocardiography skills among neonatology care providers, for both with and without prior echocardiography skills.
- This manuscript explores the role of simulators in developing echocardiography skills, their pros and cons, and how they can be utilized in training targeted neonatal echocardiography skills.

## Background

Neonatologist performed echocardiography (NPE) is rapidly becoming an essential tool to provide high quality patient care to the sick infants in the neonatal intensive care units (NICU), especially to infants with hemodynamic instability. In the last 2 decades, there has been a significant interest among the providers to learn echocardiography skills. Comprehensive guidelines for NPE and targeted neonatal echocardiography (TNE) have been published providing framework for training and quality assurance.^1–4^ Learning echocardiography is a complex process, requiring comprehensive knowledge of anatomy, physiology and technical skills in image acquisition and their interpretation.^2–4^ This process becomes even harder in neonates with a complex and dynamic physiology during the neonatal period. The cardiovascular physiology during neonatal period changes rapidly after birth and is affected by the disease or intervention to treat the sick infants with hemodynamic instability.^4,5^ In addition, there remains a significant risk of encountering undiagnosed critical congenital heart defects during the neonatal period. Hence, the guidelines for NPE recommends comprehensive training to develop echocardiography skills necessitating 6–12 months placement in a pediatric cardiology or NICU providing such specialized training. However, with the huge pressure on training resources and limited dedicated time for the neonatal fellows during their training, implementation of these guidelines remains a challenge across the world. To overcome this challenge, these guidelines have recommended the use of echocardiography simulators for the initial training.^1–4^

In this manuscript, we explore these challenges in learning echocardiography skills, role of simulators, their pros and cons, progress in developing echocardiography simulators dedicated to neonates and children, and their effectiveness.

## Learning echocardiography skills for a novice

Learning echocardiography skills is a complex task, needing a good understanding of heart anatomy, 3D spatial orientation and hand-eye coordination. A good understanding of this 3D anatomy and relationships of various parts of the heart and their interdependence needs visual orientation through videos, ideally in real time showing change in images with change of imaging planes. Experts focus on the screen and follow the structures they are interrogating on with subconscious movements of their hands, and they do not focus on their hand or probe while scanning. This can be a challenge for novice learners, and acquiring this necessary psychomotor skill and hand–eye coordination is possible only with hands-on training on a simulator or real patients.

Learning echocardiography in neonates is even harder for the following reasons: 1) complex cardiovascular physiology during the transitional period, from fetal to neonatal circulation, with rapid and significant changes fetal shunts (changes in the patent ductus arteriosus and foramen ovale) and rapid drop in in the pulmonary vascular resistance.^4,5^ These changes continue to occur during the neonatal period and are affected by the disease process, and interventions to improve the hemodynamic instability. There are drastic hemodynamic changes during the transitional period with closure of shunts, drop in pulmonary vascular resistance and increase in cardiac output^4,5^ - a detailed description of these changes is out of the scope of this chapter, 2) higher likelihood of undiagnosed or unrepaired congenital heart defects affecting normal anatomy and physiology, 3) increased risk of missing undiagnosed critical congenital heart defect if comprehensive echocardiography is not performed, 4) instability in sick infants limiting duration for scanning time, and 5) limited availability of expert supervisors.

## Why a simulator needed for neonatal echocardiography training?

Neonatologist performed echocardiography is becoming an essential tool in the modern neonatal care to gain anatomical, physiological and hemodynamic information, for understanding cardiovascular pathophysiology and to make physiology based clinical decisions at the bedside. However, developing a dedicated NPE training program remains a challenge for the vast majority of neonatal units. The authors of NPE and TNE guidelines have acknowledged the challenges neonatologists face such as how to learn echocardiography skills on vulnerable sick neonates or extremely preterm infants who can destabilize in inexperienced hands or if they are handled for longer time. Neonatologists have limited opportunities to learn echocardiography skills on actual patients and this opportunity is further hampered by the lack of adequately trained non-cardiology TNE specialists.

NPE and TNE guidelines recommend that learners are required to scan a certain number of abnormal cases, including the most important critical cardiac defects, which may become almost impossible for neonatologists receiving their training in the NICU, especially in the smaller neonatal units, and with little or no experience in working in the pediatric cardiology services.^2,4^ Echocardiography simulators with a substantial case database of variety of CHDs, and different functional / hemodynamic states have been proposed as a solution.^1–4,6–8^ Hence, NPE guidelines recommend using echocardiography training simulator to learn basic echocardiography skills – how to acquire basic echocardiography views, how to learn hand-eye movements, how to manipulate probe to acquire a particular or sweep views, and so on. Once the learners have acquired these skills on the simulator, then they can rapidly adapt and adjust to perform echocardiography on the real patients.

## Validation and effectiveness of simulators in acquisition of echocardiography skills

The efficacy of echocardiography simulators is well established in adults and anesthesiology. In a prospective randomized study on assessing the efficiency of the transthoracic echocardiography simulation to train anesthesiologists in basic transthoracic echocardiography skills, Neelankavil and colleagues randomized 61 anesthesiology residents to two groups: control (30) and simulation training in transthoracic echocardiography (31).^9^ They reported that the simulation group scored higher on all assessment criteria after the first training session: written post-test (57.9% ± 8.8% vs 68.2% ± 10.1%; *P* < 0.001), volunteer subject post-test image quality scores (0 to 25 scale) (6.4 ± 3.5 vs 12.4 ± 4.2; *P* = 0.003), anatomy identification scores (0 to 25 scale) (8.3 ± 6.6 vs 17.8 ± 6.6; *P* = 0.003), and percentage correct views (50 ± 19 vs 78 ± 21; *P* < 0.001). After the second training session, all scores were further improved in the simulation group: volunteer subject post-test image quality scores (9.6 ± 3.3 vs 15.6 ± 2.8; *P* = 0.002), anatomy identification scores: (17.6 ± 3.8 vs 22.8 2.4; *P* = 0.003), and percentage correct views (80 ± 16 vs 96 ± 8; *P* = 0.007). The authors concluded that the residents trained with simulation acquired better skills in echocardiography image acquisition and anatomy identification on volunteer subjects.^9^ In a large multicentered randomized control trial involving 324 cardiology fellows as participants, Pezel et al. demonstrated that simulation-based teaching of transesophageal echocardiography (TEE) showed a significant improvement in the knowledge, skills, and self-assessment of proficiency, as well as a reduction in the amount of time needed to complete the examination.^10^ Similarly, Matyal et al. evaluated the efficacy of probe handling and image acquisition in TEE among anesthesiologists.^11^ Initially, trainees’ progress on the echocardiography simulator was measured and then the skill transfer to real patients was assessed during a TEE examination. Trainees showed significant improvement in their manual skills after 4 weeks of simulator training.^11^ In a randomized prospective study, Edrich et al. compared efficacy of simulator training on volunteers using 46 adult physicians and anesthesiologists without prior experience in echocardiography and concluded that, for beginners without prior experience in transthoracic echocardiography, simulator training was not inferior to training using real volunteers.^12^

With the availability of dedicated neonatal and pediatric echocardiography simulators, similar strong evidence on efficacy of simulation-based training in learning echocardiography skills among neonatal fellows, neonatologists and pediatric residents has been published. In a recent multicentered randomized control trial on “effectiveness of simulation-based echocardiography training for neonatology residents”, Blanchetière and colleagues compared a control group with theoretical and bedside training, with a simulation group with theoretical training, a 3-hour simulation session and bedside training.^13^ An evaluation using the neonatal echocardiography simulator was conducted at 3 and 6 months from initial training based on two scoring methods by two evaluators: a reference score for quality of echocardiography views, and a custom-made score to assess the recognition of the anatomical structures. Among 17 controls and 35 simulation group participants, at 3 months residents in the simulation group exhibited a higher mean score for both the reference score (11.5 ± 2.3 points versus 7.4 ± 3.4 points, *P* < 0.001) and the custom-made score (25.8 ± 5.3 points versus 16.9 ± 7.8 points, *P* < 0.001) than residents in the control group. This difference between the two groups remained significant at 6 months after simulation training. The authors concluded that simulation-based training seems to be a valuable approach for echocardiography training of NICU residents and should be developed to more extensive training courses.^13^

We studied the impact of simulation training in acquiring echocardiography skills among 43 neonatal care givers (16 neonatologists, 26 neonatology fellows, and 1 nurse practitioner) with various prior exposure to echocardiography.^14^ The participants first had didactic sessions on topographical cardiac anatomy and standard echocardiographic views followed by hands-on simulator training. They were tested before and after the simulation training on their ability to obtain the 26 standard echocardiographic views and on the quality of each image on the simulator. After the hands-on simulator training, the median (interquartile) score for the quality of acquired images increased from 36 (22, 43) to 55 (48, 58), *p* < 0.0001, and the number of views with acceptable or good image quality (scores of 2 or 3) increased from 11 (6, 16) to 20 (17, 21), *p* < 0.0001. This study further supports the notion that echocardiography simulation is an effective tool in improving echocardiography skills among neonatology care providers, for both with and without prior echocardiography skills.^14^

Beyond proving the effectiveness of echocardiography simulators, there are different levels of validation of increasing value and complexity^15^: 1) Face validity: describes the realism of a simulator, 2) Content validity: describes the relevance of the simulator content for the real task and 3) Construct validity: describes the extent to which groups with more experience perform better on the simulator than groups with less real-life experience and the degree to which the results of the ‘training session’ reflect the actual skill of the trainee being assessed.^16–18^ Face validity and content validity of the neonatal echocardiography simulators have been well tested in the different clinical groups setting via standardized questionnaires.^19–23^

The construct validity of the neonatal echocardiography simulator has been tested by Weidenbach et al. on 43 participants of different expertise (beginners, intermediates and experts) on 10 cases of CHDs. They reported that experts identified almost all lesions correctly and performed significantly better than intermediates and beginners.^18^ Similar results have been produced by Noori et al. (unpublished data).

## Pros and cons of the echocardiography simulators

With the proven efficacy and validation, and increase in use of echocardiography simulators it’s important to understand their pros and cons, their strengths and weaknesses in echocardiography training, which are summarized in the table below (Table 1).

**Table 1:** Pros and cons of echocardiography training using simulators: strengths and weaknesses of providing training using echocardiography simulators is summarized.

Pros and cons of echocardiography training using simulators	
Pros	Cons
1. **Safe and flexible learning:** Flexibility in learning at convenient time – at the pace of learner, and safe learning environment 2. **Tailored made individualized learning:** Opportunity for tailor practicing as per individual’s changing needs, personalized training program with interference from clinical environment 3. **Structured training:** Structured training with desired scenarios to augment learning and independence from patient availability 4. **Established effectiveness:** Research evidence showing established effectiveness in learning echocardiography skills 5. **Cases with abnormal pathologies:** A large library of CHD cases allows recognition and evaluation of a wide spectrum of structurally abnormal hearts (congenital heart defects)	1. **Lack of patient interaction:** Lack of real challenges in patient scanning (such as patient movement, instability while scanning, adapting scanning as per patient’s position and variation in anatomy) 2. **Resources:** High acquisition costs for the high-fidelity simulators and costs of running the simulation program such as dedicated experts, space, time, etc 3. **Skills transfer to real patients:** Limited data on ‘skills transfer effectiveness’ from the simulated environment to real patients

## Neonatal and Pediatric echocardiography simulators

**Fig. 1:**
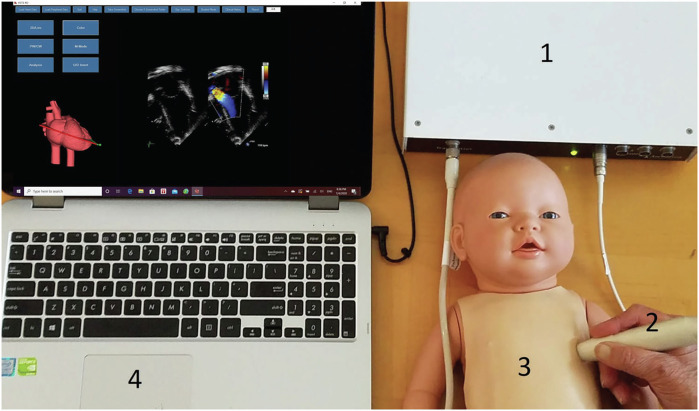
Virtual Neonatal Echocardiography Training System (VNETS): figure showing the VNETS system in use with tracking system device, mannikin, and computer.

**Fig. 2:**
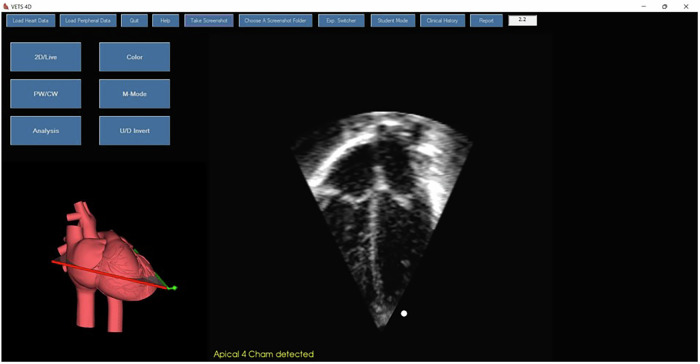
Virtual Neonatal Echocardiography Training System (VNETS): live feedback during the echocardiography simulation training. 3D heart model showing the plane of scanning while 2D image is showing the live apical 4-chamber view which is confirmed by the system (as yellow text at bottom showing 'Apical 4 chamber detected' feedback mechanism.

Echocardiography simulators are predominantly used in adult medicine. Currently there are two simulators with real images dedicated for echocardiography training in neonates and infants: 1) Virtual Neonatal Echocardiography Training Systems (VNETS, Fig. 1; www.neoechosim.com) and 2) EchoCom|Neo (www.echocom.de).^16,24^ The technical background for 2-dimensional (2D) imaging for both simulators is similar, and the efficacy of both simulators has been tested and established (as above). Both simulators use three-dimensional (3D) echocardiographic datasets acquired by a matrix probe, which are sliced into cut planes to present as 2D images (Fig. 1). The cut planes are derived from the position and orientation of a tracking sensor within an electromagnetic field, and with a dummy transducer that incorporates the tracking sensor, a manikin is examined like a real patient to get real echo images. Both simulators have display of real time 2D images and a 3D heart model on the screen. With the manipulation of the transducer, the cut plane is continuously displayed on the 3D heart model helping the trainee to optimize the desired view. In VNETS, the system feedback mechanism notifies the trainee when optimal cut plane is obtained (Fig. 2). Both systems have a large library of normal heart and congenital heart diseases. In VNETS, a background clinical history is provided for each case to give context for the indication for obtaining the echocardiogram.

In contrast to EchoCom|Neo, VNETS is a hybrid pediatric echocardiography simulator, which provides full range of pediatric echocardiography training experience. Therefore, in addition to the real time 2D imaging, VNETS is capable of simulating color flow Doppler, spectral Doppler, and M-mode echocardiography. Although the other modalities are not in real time, they enhance learning by allowing for visualization of the shunts (e.g. patent ductus arteriosus and ventricular septal defects), valvular regurgitations, flow direction and pattern and detailed motion of heart structures (m-mode). Furthermore, the hybrid mode allows for measuring velocity, distance and time, thereby enabling the trainee to assess cardiac function and measure gradients and flows. Hence, it provides all aspects of training in echocardiography what a learner needs for the comprehensive structural or functional assessment in real-life. Additionally, VNETS system doesn’t need repeated calibration during scanning as needed in the EchoCom|Neo simulator.

**Fig. 3:**
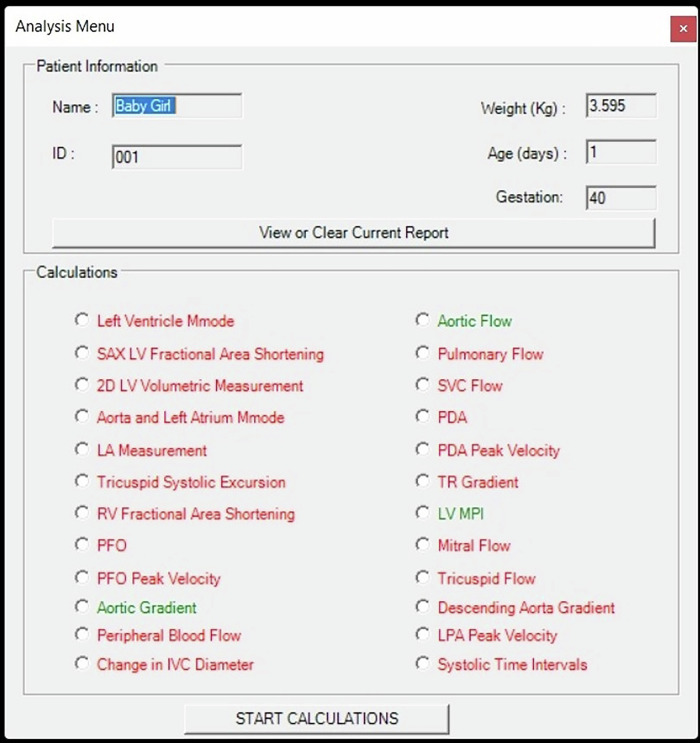
Virtual Neonatal Echocardiography Training System (VNETS): analysis options are changed depending upon the echocardiography view and what measurements can be done in that particular view – possible measurements in a particular view are visible in green while measurements which can not be done in that particular view are seen as red to help the novice learners.

Included in the analysis package of VNETS are measurements needed to assess cardiac functions such as fraction shortening, ejection fraction, Tricuspid Annular Plane Systolic Excursion (TAPSE) and fractional area change (FAC%), comprehensive hemodynamic evaluation such as measuring left and right ventricular outputs, superior vena cava flow, left atrium to aortic root ratio (LA/ Ao ratio) and inferior vena cava collapsibility index, and assessment of regional flow such as Doppler indices of celiac or superior mesenteric artery flow (Fig. 3). After completing the assessment of cardiac function and hemodynamics, the system will generate a report detailing all the measurements obtained by the operator.^25^

**Fig. 4:**
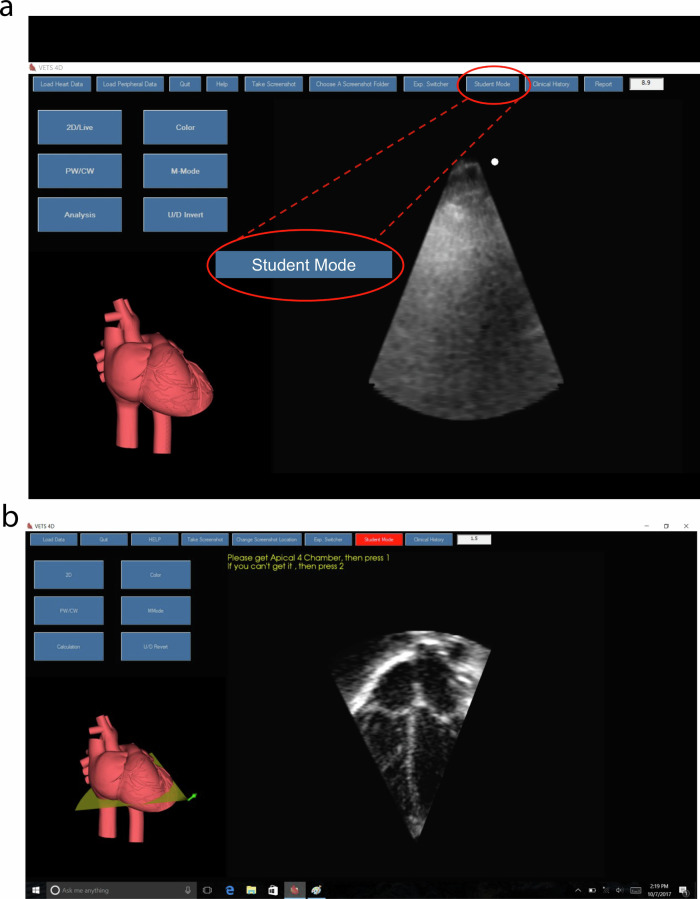
Demonstration of using student mode on the VNETS system. Figure **a** shows the selection of student mode, and figure **b** shows the instructions for the student.

Another useful feature of VNETS is the student mode (Fig. 4a, b). The trainee can choose from beginner, intermediate and advanced options. The system gives prompts to obtain specific views and after the trainee obtains each view, the system allows for saving them as still images. An integrated automated system rates the echo images for quality. This provides the trainee with instant feedback, allowing independent practice on the simulator. In addition, the saved images along with the automated quality report are available for the instructor for additional feedback and for tracking the trainee’s progress.

The VNETS has been used since 2012 in the University of Southern California annual echocardiography course for neonatologists and in other national and international workshops such Pediatric Academic Societies (PAS) and Societies for Pediatric Research (SPR) meetings and NeoHeart conferences. In addition, VNETS is now used across the world including in the USA, UK, Canada, Europe, Australia, Africa and South America where even the personnel with good echocardiography skills find it very useful, especially for learning how to a comprehensive hemodynamic evaluation or pattern recognition of abnormal cases, and in maintenance of echocardiography skills in recognition of rarer congenital heart defects.

## Collection of cases in VNETS simulator

VNETS has excellent collection of interesting cases varying from normal heart to anatomical and hemodynamic pathologies such as hemodynamically significant large patent ductus arteriosus (PDA), pericardial effusion, pulmonary hypertension/persistent pulmonary hypertension of the newborn (PPHN), changes during transitional circulation, hypovolemia, neonatal shock, and all the common congenital heart defects such as transposition of great arteries (TGA), total anomalous pulmonary venous connections (TAPVC), tetralogy of Fallot (TOF), pulmonary atresia (PA), tricuspid atresia, truncus arteriosus, coarctation of aorta (COA), hypoplastic left heart, isomerism, dextrocardia, etc. – just to name a few.

While the common neonatal cases such PDA, PPHN, neonatal shock allow the learners to practice all echocardiography skills needed for a comprehensive hemodynamic evaluation, the common CHD cases help the learners in pattern recognition if they encounter them later in their practice and learn about the abnormalities.

## Future Direction – Research, Training and Simulators Development

Integration of point-of-care ultrasonography (POCUS) in clinical practice is well established as part of standard of care and various transferred based protocols have been developed for use in adults and older children. However, use of POCUS is relatively new in neonates although its popularity has been rapidly increasing after publication of “international evidence-based POCUS guidelines for use in neonates and children”.^26,27^ The application of ultrasonography in neonates is harder when it comes to point-of-care echocardiography because of longer duration of training and limited resources. Hence, the role of simulators providing real cuts simulating real echocardiography in various congenital heart defects cases and hemodynamic abnormalities becomes even more crucial in neonates.^1,2,28^ The VNETS system provides excellent artificial intelligence (AI) guided scoring on the quality of images in the ‘student mode’ which can help the learners to observe their progress in imaging acquisition. In addition, the learners can test their abilities to make the clinical diagnosis in various case scenarios. There is a need to make this automated learning even more effective and develop tools to help learners monitor their progress. The currently available simulators facilitate learning of this complex skill, but further simulation development is needed to make it even more interactive. The current high-fidelity simulators are expensive, which pose a barrier to a more widespread adoption in echocardiography training programs. Advances in tracking systems technology and increasing competition may make echocardiography simulation more affordable and accessible in future. Further research should focus on effectiveness of simulators in different clinical setting and on leaners ability to transfer the ‘skill set’ from the simulation environment to the real patient settings.

## Conclusion

Neonatologist performed echocardiography is becoming an essential tool to deliver high quality patient care, especially those infants with hemodynamic instability and those needing serial echocardiography assessments. There is ample evidence that NPE improves clinical decision making in the NICU. However, with the limited opportunities, learning echocardiography skills, especially on the sick neonates and infants, remains a big challenge. Various guidelines have proposed frameworks for learning echocardiography skills and they provide excellent training resources for the learners. These guidelines recommend using simulator to learn echocardiography skills, especially in the beginning of training and for pattern recognition of cases which are not commonly seen in the neonatal units. Simulators dedicated to learning skills in targeted neonatal echocardiography are now available and they have been validated as an effective tool in improving echocardiography skills among neonatology care providers, for both with and without prior echocardiography skills.

## Data Availability

This is a narrative review article, so the data availability is not applicable here.
